# Pituitary Adenylate Cyclase-Activating Polypeptide (PACAP) in Physiological and Pathological Processes within the Gastrointestinal Tract: A Review

**DOI:** 10.3390/ijms22168682

**Published:** 2021-08-12

**Authors:** Aleksandra Karpiesiuk, Katarzyna Palus

**Affiliations:** Department of Clinical Physiology, Faculty of Veterinary Medicine, University of Warmia and Mazury in Olsztyn, Oczapowskiego Str. 13, 10-718 Olsztyn, Poland; aleksandrakarpiesiuk@wp.pl

**Keywords:** gastrointestinal tract, PACAP, mammals, pathological conditions, physiology

## Abstract

Pituitary adenylate cyclase-activating polypeptide (PACAP) is a neuropeptide widely distributed in the central nervous system (CNS) and many peripheral organs, such as the digestive tract, endocrine, reproductive and respiratory systems, where it plays different regulatory functions and exerts a cytoprotective effect. The multifarious physiological effects of PACAP are mediated through binding to different G protein-coupled receptors, including PAC1 (PAC1-R), VPAC1 (VPAC1-R) and VPAC2 (VPAC2-R) receptors. In the gastrointestinal (GI) tract, PACAP plays an important regulatory function. PACAP stimulates the secretion of digestive juices and hormone release, regulates smooth muscle contraction, local blood flow, cell migration and proliferation. Additionally, there are many reports confirming the involvement of PACAP in pathological processes within the GI tract, including inflammatory states, neuronal injury, diabetes, intoxication and neoplastic processes. The purpose of this review is to summarize the distribution and pleiotropic action of PACAP in the control of GI tract function and its cytoprotective effect in the course of GI tract disorders.

## 1. Introduction

Pituitary adenylate cyclase-activating polypeptide (PACAP) is a neuropeptide that was first identified in an ovine hypothalamus extract in 1989. It is involved in stimulating adenylate cyclase from cultured rat pituitary cells [1]. PACAP is encoded by the ADCYAP1 gene located on chromosome 18, which has four exons. Exon 4 encodes PACAP and originates two biologically active peptide isoforms [2]. PACAP-38-consisting of 38 amino acids and is C-terminally α-amidated. PACAP-27-is the result of post-translational processes in which it is shortened at the C-terminus, keeping the same amino acid sequence at the N-terminus [2,3]. Previous studies have shown that PACAP-38 is the dominant form in mammals [3]. PACAP exhibits a high degree of homology with vasoactive intestinal peptide (VIP) on the N-terminus amino acid sequence (68%) and belongs to the VIP/glucagon/secretin superfamily [4]. Amino acid sequences of the members of this superfamily were described by Vaundry et al. [5]. The occurrence of PACAP was confirmed in the central nervous system (CNS) and many peripheral organs, such as the digestive tract, endocrine, reproductive, and respiratory systems, where it plays different regulatory functions and exerts a cytoprotective effect [4,5,6,7,8]. In the gastrointestinal (GI) tract, PACAP plays an important regulatory function. PACAP stimulates the secretion of digestive juices and hormone release, regulates smooth muscle contraction, local blood flow, cell migration and proliferation [8,9,10]. Additionally, there are many reports confirming the involvement of PACAP in pathological processes within the GI tract, including inflammatory states, neuronal injury, diabetes, intoxication, and neoplastic processes [10,11,12,13,14,15]. Therefore, the purpose of this review is to summarize the distribution and pleiotropic action of PACAP in the control of GI tract function and its cytoprotective effect in the course of GI tract disorders.

## 2. Localization of PACAP in the Gastrointestinal Tract in Individual Species of Mammals

The presence of PACAP in the GI tract has been described in various mammal species, including humans [7,10,11,16,17,18,19,20,21,22,23,24,25,26,27,28,29,30,31,32,33]. However, it should be emphasized that the distribution of PACAP differs between different parts of the GI tract in the same species and shows interspecies differences. The studies on the distribution of PACAP in the GI tract in individual mammalian species are presented below and summarized in Table 1.

### 2.1. Rodents

Numerous PACAP-immunoreactive (IR) neurons and nerve fibers have been found in the myenteric and submucous plexuses in the rat small and large intestine [16]. The presence of PACAP-IR fibers was described in the circular and longitudinal muscle layers using both light and electron microscopy. Additionally, a synaptic connection of PACAP-IR cell bodies with PACAP-containing nerve fibers was also demonstrated [9]. Detailed distribution of PACAP in the rat digestive tract (esophagus, stomach, small and large intestine) was demonstrated using radioimmunoassay, chromatography, immunocytochemistry, and in situ hybridization by Hannibal et al. [7]. Research has shown that PACAP-38 is the predominant form in this species. Fibers immunoreactive to PACAP have been described in both layers of muscularis as well as in the mucosa in the entire length of the GI tract and in both types of enteric ganglia (submucous and myenteric). However, PACAP-IR cell bodies have been visualized in the myenteric ganglia in the esophagus, stomach, small and large intestine and submucous ganglia only in the small intestine. PACAP is also present in the enterochromaffin-like (ECL) cells of the gastric mucosa [17]. PACAP and its mRNA was also detected in the pancreas and the gastric mucosa using the sandwich enzyme immunoassay (S-EIA) and RT-PCR technique [18].

In the available literature, there are few reports describing the distribution of PACAP in the GI tract of mice. The presence of PACAP-IR nerve fibers has been described in the myenteric ganglia and smooth muscle in the gut. Additionally, PACAP-IR single nerve fibers were visualized in the gastric mucosa [19]. Other authors visualized PACAP only in the circular muscle layer of the wild-type mouse antrum [20].

PACAP immunoreactivity was also studied in guinea pig small and large intestines. Fibers were mainly detected in the myenteric and submucous plexuses, in the longitudinal and circular muscle layers and around blood vessels of the submucosa. PACAP-positive neurons were detected only in myenteric ganglia and, due to their morphology, were qualified for Dogiel type-I [21]. In another study in guinea pig, immunolabeled nerve fibers were described in both myenteric and submucous ganglion in the jejunum with a significant numerical advantage in the myenteric ganglion. Similarly, PACAP-containing nerve fibers were visualized in both enteric ganglia [22].

### 2.2. Pigs

The distribution of PACAP in neuronal structures within the GI tract of the pig is well documented. PACAP-IR nerve terminals have been visualized in the striated muscle of the porcine esophagus [23]. In the prepyloric area of the porcine stomach, PACAP-IR neurons have been identified in both submucous and myenteric plexuses, where they constituted only a small percentage of enteric neurons [24]. A similar observation was made in the porcine corpus of the stomach [11]. A slightly larger, but still not very numerous, population of PACAP-immunopositive enteric neurons has been described in all type of enteric plexuses (i.e., myenteric plexus (MP), outer submucous plexus (OSP), and inner submucous plexus (ISP)) within the small intestine (duodenum, jejunum, and ileum) [11].

The occurrence of PACAP in intramural ganglia in the duodenum has also been described by other authors [25,26]. Additionally, Gonkowski et al. [12] visualized a moderate number of PACAP-IR fibers in the porcine ileum. The distribution pattern of PACAP was also determined in the porcine descending colon. PACAP-IR cell bodies were present in enteric plexuses (MP, OSP and ISP), and PACAP-immunopositive fibers were detected in enteric plexuses, the circular muscle layer and the mucosal layer [10,11].

### 2.3. Humans

Similar to the pig, PACAP has been detected along the entire length of the human digestive tract. According to Uddaman et al. [27], single PACAP-positive neurons and a dense network of PACAP-IR intraganglionic nerve fibers have been detected in the myenteric ganglia in the lower esophagus. Moreover, abundant PACAP-containing nerve fibers were observed in the longitudinal and circular muscle layers. Further studies performed on the stomach confirmed the presence of low-density nerve fibers in both the mucosa and the muscle layers, numerous PACAP-containing cells in the glands of the fundus and the corpus and slightly less numerous in the cardiac and pyloric glands [28]. Rare PACAP-IR nerve profiles were also described in the human stomach by Sundler et al. [19]. In the small intestine, numerous PACAP-immunopositive nerve fibers were detected in all intestinal layers and in the enteric ganglia. In turn, PACAP-IR cell bodies were abundant in the submucous ganglia and slightly less numerous in the myenteric ganglia within the small intestines [19]. Within the large intestine, the most detailed study on the distribution of PACAP in the enteric nervous system was made by Godlewski and Łakomy [14]. It was found that the number of PACAP-IR neuronal cells in the submucous plexus in the colon amounts to about 45%, while in the myenteric plexus, PACAP was noted in about 32% of all enteric neurons. In both enteric plexuses, numerous PACAP-IR fibers were also observed. Other studies have reported a similar distribution of PACAP in neuronal structures of ENS [29]. Moreover, PACAP-containing fibers were observed in the mucosa and muscular layers [29,30,31].

### 2.4. Other Species

There are also reports describing the presence of PACAP in the wall of the GI tract of cat, sheep and ferret [19,28,32,33]. In all species, PACAP-immunopositive nerve fibers were observed in the myenteric ganglia and muscle layers in the entire length of the digestive tract [19]. More detailed research was conducted on sheep. Köves et al. [33] described the presence of PACAP in nerve fibers in the longitudinal muscle layer in the esophagus, stomach, and small and large intestines, the circular muscle layer of the stomach and intestine and the arterial walls in the duodenum. PACAP-containing fibers was also observed in the pancreas, including islets of Langerhans and the small arteries. Moreover, PACAP-IR cell bodies were described in the intrapancreatic ganglia, in which PACAP-positive cell bodies accounted for about 88% of all intrapancreatic neurons [32]. In turn, in cats, the distribution of PACAP was examined in detail in the stomach. PACAP-IR cells were visualized in the stomach glands, especially in the corpus, and numerous PACAP-immunopositive fibers were observed in the mucosa and the muscle layers [28].

## 3. Co-Localization of PACAP with Other Neuroactive Substance in the GI Tract

The ratio of colocalization of PACAP with other neuroactive substance in the neuronal structures in the wall of the GI tract was also determined using the double-immunofluorescence technique in many mammalian species. Generally, in the esophagus, PACAP co-localized with VIP in both enteric neurons and nerve fibers [7,27]. Moreover, in the myenteric plexus, PACAP co-existed with neuropeptide Y and helospectin [7,9]. In the stomach, PACAP colocalized with VIP, calcitonin gene-related peptide (CGRP) and gastrin-releasing peptide (GRP) in the nerve fibers in both the mucosa and muscular layers [7]. However, in PACAP-IR, myenteric neurons were also VIP-, GRP-and nNOS-positive [7,28]. Similarly, in the small intestine, numerous PACAP-IR neurons and nerve profiles in all intestinal layers were simultaneously immunoreactive to VIP [7,22]. PACAP co-localized with nNOS only in the myenteric neurons and nerve fibers in the muscle layers. Only single perivascular PACAP-IR fibers were also CGRP-positive [7]. In turn, in the large intestine, the co-existence of PACAP with VIP, nNOS and the cocaine-and amphetamine-regulated peptide transcript (CART) was only observed in the nerve fibers in all intestinal layers, while PACAP+/CGRP+ nerve profiles were visualized only in the mucosa [7,34].

## 4. PACAP Receptors and Their Localization in the GI Tract

**Figure 1:**
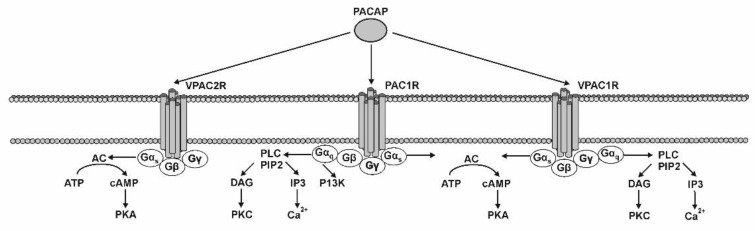
PACAP receptors (PAC1R, VPAC1R, and VPAC2R) and their biological action. cAMP-cyclic adenosine3′,5′-monophosphate; AC-adenylate cyclase; PKA-protein kinase A; ATP-adenosine triphosphate; Gα/β/γ-G protein alpha/beta/gamma subunit; PLC-phospholipase C; PIP2-phosphatidylinositol 4,5-bisphosphate; DAG-diacylglycerol; IP3-inositol trisphosphate; PI3K-phoshoinositide 3-kinase. The figure is based on the graphics contained in the article of Gabriel et al. [40].

The multifarious physiological effects of PACAP are mediated through binding to different G protein-coupled receptors, including PAC1 (PAC1-R), VPAC1 (VPAC1-R) and VPAC2 (VPAC2-R) receptors [35] (Figure 1). PAC1-R exerted a 100-fold higher affinity for PACAP than for VIP, whereas VPAC1-R and VPAC2-R exhibit a comparable affinity to PACAP and VIP [36]. Molecular studies identified diverse receptor conformational ensembles and microstate transition paths for each receptor and revealed differential peptide-receptor interactions (at the atomistic detail) for each receptor important for PAC1, VPAC1 and VPAC2 receptor ligand selectivity [37].

The PAC1-R has various variant transcripts (Null, Hip, Hop1, Hop2, Hiphop1, Hiphop2, short and very short isoforms), which lead to the activation of two different signaling pathways: increasing the intracellular level of cyclic AMP (cAMP) by adenylyl cyclase (AC) and the stimulation of phospholipase C (PLC) [36,38]. Activation of VPAC1-R and VPAC2-R stimulate AC and some other cAMP-independent signaling cascades [39].

To date, the distribution of PAC1-R in the GI tract has been confirmed in the smooth muscle of the stomach in guinea pig [41] and neuroendocrine cells (enterochromaffin-like cells (ECL)) in rats [17,42], the gastric artery in dogs [43], the smooth muscle of ileum in guinea pigs [44], rats [45], and dogs [46], the longitudinal smooth muscle of the colon in humans [47], and rats [48], the circular smooth muscle of the colon in guinea pigs [49], taenia caeci in guinea pigs [50], as well as in human and rat livers [51,52], the guinea pig gallbladder [53] and the rat pancreatic acinar cell line AR 4-2J [54]. Additionally, the expression of PAC1-R was detected on the myenteric neurons located in the human GI tract [55] and rat gastric and colonic myenteric neurons [56].

VPAC1-R was also widely distributed in the digestive tract. The expression of VPAC1-R has been detected in the human intestinal cell lines [57], the internal anal sphincter in opossum [58], jejunum, ileum and colon in mice [59], sigmoid colon and small intestines in humans [55,59], jejunum in mice [60], pancreas islets in rats [61] and insulin-secreting cell line MIN6 in mice [62].VPAC2-R was most commonly found in neuroendocrine cells, blood vessels and smooth muscle in the human small intestine [55], smooth muscle of the stomach in rabbits [63], guinea pigs [64] and rats [65], taenia coli in guinea pigs [64], the pancreas in rats [61] and insulin-secreting cell line MIN6 in mice [62]. Additionally, there are reports describing the expression of common PACAP/VIP receptor along the entire length of the digestive tract (esophagus, stomach, small intestines, colon, liver and pancreas) in several animal species without a clear distinction between type 1 and type 2 [60,65,66]. Both VPAC1-and VPAC2-receptors were also detected in the submucous plexus of the mouse jejunum [60].

## 5. Physiological Role of PACAP in the GI Tract

**Figure 2:**
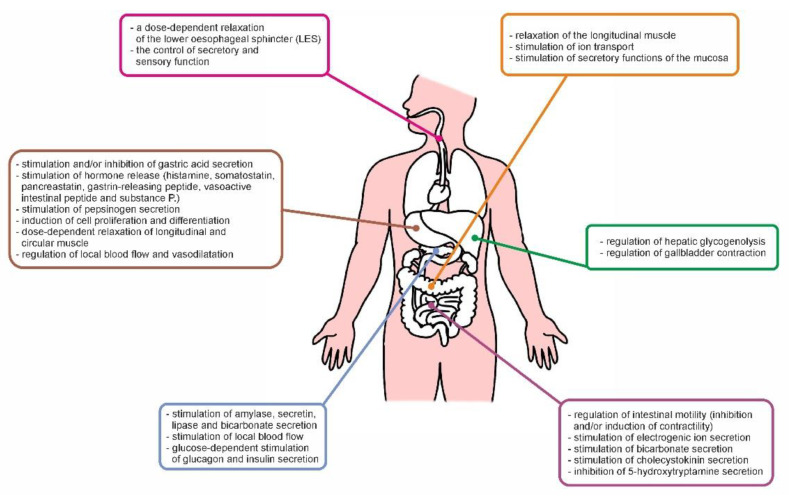
Summary of physiological effects of PACAP in the GI tract.

There are many reports confirming the significant role of PACAP in the control of the physiological functions of the mammalian GI tract [8,9,41,42,43,53,66,67,68,69,70,71,72,73,74,75,76,77,78,79,80,81,82,83,84,85,86,87,88,89,90,91]. Generally, PACAP regulates secretion, motility, blood flow and proliferation, but its effect depends on both the part of the GI tract and stimulated receptors (Figure 2). In the esophagus, PACAP evokes a dose-dependent relaxation of the lower esophageal sphincter (LES) in human and cats [66]. There are also reports suggesting the involvement of PACAP in the control of secretory and sensory function in this part of the GI tract [42].

Moreover, several studies have shown that PACAP stimulates gastric acid secretion and hormone release in the stomach. Regulation of secretory functions is accomplished by PACAP through interaction with neuroendocrine cells. The presence of the PAC1 receptor on ELC cells and the common receptors for VIP/PACAP on D cells was demonstrated [67]. The vast majority of available data confirm that PACAP directly induces hydrochloric acid secretion and stimulates histamine secretion [68,69]. In contrast, some authors demonstrated that PACAP inhibits gastric acid secretion by stimulation of somatostatin release from D cells [70]. PACAP probably stimulates the secretion of hydrochloric acid immediately after filling the stomach with a meal (the neural phase) and inhibits it in the later phase by increasing the secretion of somatostatin [71]. Furthermore, PACAP stimulates the release of other substances in the stomach, such as pancreastatin, gastrin-releasing peptide, VIP, and substance P (SP) [8]. There are also reports describing the stimulatory effect of PACAP on pepsinogen secretion from isolated chief cells in the guinea pig [67]. As mentioned above, PACAP induces cell proliferation and differentiation. PACAP, as a strong mitogen, stimulates the proliferation of ECL cells with up to 100 times stronger potency than VIP and higher efficiency than gastrin [72]. However, research by van Assche et al. [73] on primary explant cultures of rabbit gastric antrum smooth muscle revealed that PACAP does not affect myocyte proliferation. One of the most detailed described effects of PACAP in the stomach is the regulation of smooth muscle contractility. PACAP exerts a dose-dependent relaxation of longitudinal and circular muscle strips of the fundus in many mammal species, including rats, mice, guinea pigs and rabbits [41,65,74,75]. PACAP also elicits an inhibitory effect on the spontaneous phasic contractions of the pylorus via activation of common VIP/PACAP receptors and, thus, regulates gastric emptying [53]. Additionally, due to the presence of PACAP in the nerve fibers innervating blood vessels, the vasodilatory role and regulation of local blood flow were also confirmed. In particular, PACAP exhibits a strong vasodilatory effect in the left gastric artery in dogs via acting on PAC1 receptors [43].

In the case of the small intestine, the regulation of intestinal motility is considered the most important physiological role of PACAP. Previous studies have shown that its effect depends on the animal species, the site of action and stimulated receptor. In vitro studies on the isolated rat ileum showed that PACAP elicits intestinal smooth muscle relaxation, and this effect is many times higher than VIP [45,75]. In turn, in the guinea pig ileum, it induced contractility, mainly by the release of acetylcholine and substance P [41,44]. A similar observation was made by Onaga et al. [76] in the ovine duodenum. Recent studies have shown that PACAP is one of the major neurotransmitters of the enteric inhibitory motor neurons (IMNs) located in the myenteric plexus and exhibiting a relaxing effect on the muscle the gut circular [77]. Furthermore, PACAP plays an important role in the regulation of intestinal secretion. PACAP stimulated electrogenic ion secretion in the jejunum [78] in rats and humans [79]. PACAP is also a potent stimulator of bicarbonate secretion in the rat duodenum [80]. There are also reports describing the stimulatory effect of PACAP on cholecystokinin (CCK) and secretin secretion and inhibition of 5-hydroxytryptamine (5-HT) in the rat and mouse small intestine [81,82,83].

Knowledge of the physiological role of PACAP in the large intestine is more fragmentary. Most of the available data concern the human colon [9,47]. PACAP induced relaxation of the longitudinal muscle of human sigmoid colon and rat distal colon and guinea pig caecum in vitro [47,48,84]. It is also likely that PACAP stimulates ion transport and the secretory functions of the mucosa [9].

In the liver, due to the induction of glucose output from the perfused rat liver by PACAP, the latter is regarded as a regulator of hepatic glycogenolysis [85]. There also reports describing its influence on gallbladder contraction. The stimulatory effect was probably mediated by activation of PAC1R, whereas activation of VPAC receptors led to a relaxation of smooth muscle contraction [53]. In turn, in the pancreas, PACAP is one of the most potent secretory agents and stimulates both exocrine and endocrine secretion. It was confirmed that PACAP induces amylase [86], secretin, amylase and lipase [87,88] and bicarbonate secretion [89] and increases local blood flow [90]. The influence of PACAP on pancreatic endocrine secretion has been studied in detail in many in vitro and in vivo animal models and humans. PACAP participates in glycemic control by glucose-dependent stimulation of glucagon and insulin secretion [90,91].

## 6. PACAP Participation in Pathological Processes in the GI Tract

An increasing number of reports have confirmed the involvement of PACAP in the regulation of pathological processes in the GI tract. PACAP has been shown to have anti-inflammatory, antioxidant and cytoprotective effects [7,10,11,12,13,14,15,29,30,31,92,93,94,95,96,97,98,99,100,101,102,103,104,105,106,107,108,109]. The involvement of PACAP in the regulation of gastrointestinal disorders is presented below and summarized in Table 2.

### 6.1. Inflammatory Condition

The anti-inflammatory properties of PACAP have been observed in acute ileitis in mice [92]. Mice infected with Toxoplasma gondi treated simultaneously with synthetic PACAP showed increased anti-inflammatory IL-4 concentration in mesenteric lymph nodes, a greater density of small intestinal FOXP3+ cells and reduced level of pro-inflammatory cytokines (IL-23p19, IL-22, IFN-γ, MCP-1) resulting from a decreased number of ileal leucocytes. In addition, PACAP-treated mice had a higher survival rate than placebo-treated mice [92]. Similarly, in experimentally-induced subacute ileitis in mice, PACAP elicited a cytoprotective effect on ileal epithelia and decreased the level of T lymphocytes, which resulted in a reduced synthesis of pro-inflammatory cytokines in the intestinal wall [93]. Furthermore, Illes et al. [94] investigated the effect of PACAP on intestinal INT407 culture cells exposed to different species of bacteria. Although PACAP had no impact on the number of bacterial colonies and adhesion, it led to a reduction in IL-8 and CXCL-1 secretion in INT407 cells. Other studies have shown that PACAP deficient mice exhibits altered intestinal microbiota composition and the complete absence of bifidobacteria, which may predispose them to increased frequency of intestinal disorders [95].

There are also many reports describing the beneficial effect of PACAP in the course of large intestine inflammation. Increased level of PACAP mRNA was observed in dextran sulphate sodium (DSS)-induced colitis in mice [96]. Other authors using the DSS-induced model of colitis have shown that PACAP-deficient mice had a reduced level of pro-inflammatory cytokines in the proximal and distal colon and more severe clinical symptoms of colitis [97,98]. Furthermore, in the acute Campylobacter jejuni-induced enterocolitis in mice PACAP treatment led to reduction of clinical symptoms such as wasting and diarrhea and less severe the microscopic features of colitis [99]. Moreover, Gonkowski and Całka [10] reported that both chemically-induced inflammation and proliferative enteropathy led to an increased population of PACAP-IR enteric nerve cells and fibers in the wall of the porcine descending colon. In comparison, patients with inflammatory bowel disease exhibited higher expression of PACAP, which was reversed by the administration of antibiotics [100]. Symptomatic diverticular disease in human resulted in an upregulated level of PACAP in the enteric plexuses in the colonic mucosa [30]. In turn, a reduced density of PACAP-containing nerve fibers was observed in the colon mucosa in the course of drug-resistant ulcerative colitis in children, which may be a result of the degeneration of mucosa [31].

### 6.2. Injuries and Intoxications

To date, numerous studies on the cytoprotective role of PACAP in the GI tract and its role in recovery processes after damage have been conducted. Nedvig et al. [101] demonstrated that PACAP shows a cytoprotective and anti-inflammatory role in the rat intestinal autotransplantation model. Further research has shown that PACAP protects the intestinal structure and alleviates the oxidative stress associated with intestinal damage during ischemia-reperfusion and autotransplantation [102]. Moreover, PACAP-38 knockout mice showed pathological changes in the intestine, including the destruction of the mucosal layer and crypts, increased tissue lipid peroxidation after both cold and warm preservation in the course of small intestinal transplantation [103]. The protective role of PACAP on intestinal epithelial cells having high turnover (INT 407) against oxidative stress was also demonstrated [104]. Recent research elucidated that PACAP preserves mitochondrial functionality and suppress apoptotic signaling in oxidative stress condition [105]. In turn, in atrophic rat ileum, decreased expression of PACAP was reported, as well as a transient supersensitivity of the longitudinal muscle to the PACAP [106]. Extrinsic denervation decreased the concentration of PACAP in the wall of the rat stomach but had no influence on the small intestine [7]. In contrast, an increased population of PACAP-IR cell bodies in each kind of enteric plexuses and a higher density of PACAP-containing nerve fibers in the mucosa and muscle layers of the descending colon was observed in pigs subjected to axotomy of caudal colonic nerves [10]. This may be explained by the different role of PACAP in the pathophysiological processes in different animal species and in particular sections of the digestive tract.

There are also reports describing the engagement of PACAP in the control of stomach hyperacidity, which may lead to mucosal barrier damage, ulcers and cancer. Elevated PACAP-immunoreactivity was observed in the submucous plexuses within the porcine stomach after experimentally induced hyperacidity [24]. In a rat model of ulceration, a higher density of PACAP-IR nerve fibers was observed in the smooth muscle adjacent to the ulcer and an upregulated level of PACAP mRNA was detected in the myenteric ganglia [107].

The latest research focuses on the role of PACAP in the protection of the gastrointestinal tract against the effects of toxins and drugs. Czajkowska et al. [25,108] investigated the effect of non-steroidal anti-inflammatory drugs (NSAIDs) on the neurochemical phenotype of enteric neurons in the pig duodenum. Both naproxen and indomethacin administration led to a significant increase in PACAP-immunoreactivity in all intramural plexuses in the duodenum as a result of mucosa damage. Additionally, bisphenol A dietary exposure resulted in an increased density of PACAP-positive hepatic nerve fibers in the pig [13]. A higher number of PACAP-contained nerve fibers within the circular muscle layer of the porcine ileum was also noted in the course of zearalenone intoxication [12].

### 6.3. Neoplastic Processes

As a cytoprotective factor, PACAP plays an important role in maintaining organism homeostasis. However, it can also participate in oncogenesis, leading to the growth of tumors located in the intestines. The distribution of PACAP was also determined in the human tumors located in the large intestine. Decreased PACAP-immunoreactivity was determined in colon tumor samples by radioimmunoassay [15]. Less numerous PACAP-IR nerve fibers in the submucosal and myenteric plexuses in sections with cancer of the human large intestine were also established [14]. Furthermore, Le et al. [109] demonstrated the presence of the PAC1 receptor on HCT8 human colonic tumor cell and the beneficial effect of PACAP on cell viability and regulating Fas-R expression, which suggests the involvement of PACAP in colon cancer development.

### 6.4. Other Disorders of the GI Tract

The occurrence of PACAP-containing nerve structures was determined in ganglionic and aganglionic portions of the large intestine of patients with Hirschsprung’s disease. PACAP was present in all intestinal layers, but a small number of PACAP-IR nerve fibres were visualized in aganglionic segments of the intestine [29]. In turn, elevated PACAP immunoreactivity in the neuronal cell within the porcine digestive tract was demonstrated in the course of diabetes [11]. Prolonged hyperglycemia led to a significant increase in the population of PACAP-IR neurons in all types of intramural neurons in the stomach, duodenum, jejunum, ileum and descending colon. The severity of the changes depended on the examined plexus and gastrointestinal segment. Nevertheless, the obtained results confirm the participation of PACAP in regulatory processes of the GIT function in the course of diabetes.

## 7. Therapeutic Use of PACAP and Its Receptors Agonists

The main problem in the therapeutic use of PACAP is its restricted bioavailability and rapid degradation (a plasma half-life less than 5 min) [110]. Due to the fact that most of the beneficial properties of PACAP are mediated via PAC1-R, recent structure-function and conformations studies have focused on finding a selective agonist for the PAC1-R with less affinity for the VPAC1 and VPAC2 receptors [110,111]. Conformational analysis of PACAP-27 has shown an initial disordered N-terminus sequence of eight amino acid residues followed by a region, from amino acid residues 9 to 24, that consists of four distinct domains [112]. The first domain (residues from 9 to 12) forms a *β*-turn-like conformation whereas the three others are composed of distinct helical regions that extend from residues 12 to 14, 15 to 20, and 22 to 24, respectively [5]. The conformation of PACAP-38 is the same as PACAP-27 in the N-terminal region (region 1–27) and shows slight differences in the C-terminal region, which suggests that the N-terminal region of PACAP is responsible for its biological activity [5,113]. The three-dimensional structure of PACAP shows a high degree of similarity to that of other VIP/glucagon/secretin superfamily members, particularly VIP. However, there are slight conformational differences between VIP and PACAP resulting in differences in the selectivity of the peptides for their receptors [5,112]. The discovery of a synthetic metabolically stable analogue of PACAP (acetyl-[Ala15, Ala20]PACAP38-propylamide), a super-agonist towards the PAC1-R and other PAC1-R selective agonists created potential therapeutic opportunities in some clinical conditions [110,111]. However, PAC1-R has numerous splice-variants with different affinities for PACAP (and its analogues) and abilities to activate intracellular response [110]. Future research will focus on the distribution of individual receptor splice-variants in tissues/organs and the therapeutic effects of individual analogs in these tissues.

## 8. Conclusions

Numerous morphological and neurochemical studies indicated that PACAP is widely distributed in the GI tract of numerous species, including humans. PACAP participates in many physiological functions in the digestive tract, such as regulation of motility, the secretion of digestive juices, exocrine function of the pancreas, intestinal absorption, cell migration and proliferation. Moreover, an increasing number of scientific reports have confirmed that PACAP is an important cytoprotective factor with anti-apoptotic, anti-inflammatory and antioxidant properties. Recent research has also demonstrated that PACAP is involved in the control of inflammatory states, recovery processes after neuronal damage, intoxication and neoplastic processes in different segments of the GI tract and various animal species. The discovery of a synthetic metabolically stable analogues of PACAP may contribute to its application in the treatment of gastrointestinal disorders, which requires further research.

## Figures and Tables

**Table 1:** Distribution of PACAP in the GI tract of various mammals’ species.

Species	Localization	References
**Oesophagus**		
Rat	nerve fibers in the circular and longitudinal muscle layers, myenteric and submucous plexuses and in the mucosa	[7]
Pig	nerve terminals in the striated muscle	[23]
Human	neurons in the myenteric ganglia intraganglionic nerve fibers in the myenteric ganglia nerve fibers in the circular and longitudinal muscle layers	[27] [27] [28]
Cat	nerve fibers in the myenteric ganglia and muscle layers	[19]
Sheep	nerve fibers in the myenteric ganglia and muscle layers	[19,33]
Ferret	nerve fibers in the myenteric ganglia and muscle layers	[19]
**Stomach**		
Rat	nerve fibers in the circular and longitudinal muscle layers, myenteric and submucous plexuses and in the mucosa neurons in the myenteric ganglia the enterochromaffin-like (ECL) cells in the mucosa	[7,18] [7,18] [17]
Mouse	nerve fibers in the mucosa nerve fibers in the circular muscle layer in the antrum	[19] [20]
Pig	neurons in the myenteric and submucous plexuses in the corpus and prepyloric area	[11,24]
Human	nerve fibers in the mucosa and muscle layers cells in the gastric glands	[19] [28]
Cat	nerve fibers in the myenteric ganglia, muscle layers and mucosa cells in the gastric glands	[19,28]
Sheep	nerve fibers in the myenteric ganglia and muscle layers	[19,33]
Ferret	nerve fibers in the myenteric ganglia and muscle layers	[19]
**small intestine**		
Rat	neurons in the myenteric and submucous plexuses nerve fibers in the circular and longitudinal muscle layers, myenteric and submucous plexuses and in the mucosa	[7,9,16]
Mouse	nerve fibers in the myenteric ganglia and smooth muscle	[19]
Guinea pig	nerve fibers in the circular and longitudinal muscle layers, myenteric and submucous plexuses and around blood vessels of the submucosa neurons in the myenteric ganglia	[21,22]
Pig	neurons in the myenteric plexus, outer submucous plexus, and inner submucous plexus nerve fibers in the mucosa and both muscular layers in the ileum	[10,11,12,25,26]
human	neurons in the myenteric and submucous ganglia nerve fibers in the circular and longitudinal muscle layers, in the mucosa and in the enteric ganglia	[19]
Cat	nerve fibers in the myenteric ganglia and muscle layers	[19]
Sheep	nerve fibers in the myenteric ganglia and muscle layers and arterial walls	[19,33]
Ferret	nerve fibers in the myenteric ganglia and muscle layers	[19]
**large intestine**		
Rat	neurons in the myenteric and submucous plexuses nerve fibers in the circular and longitudinal muscle layers, myenteric and submucous plexuses and in the mucosa	[7,9,16]
Guinea pig	nerve fibers in the circular and longitudinal muscle layers, myenteric and submucous plexuses and around blood vessels of the submucosa neurons in the myenteric ganglia	[21,22]
Pig	neurons in the myenteric plexus, outer submucous plexus, and inner submucous plexus in the descending colon nerve fibers in the circular and longitudinal muscle layers, in the mucosa and in the enteric plexuses	[10,11]
Human	neurons in the myenteric plexus and submucous plexus nerve fibers in both enteric plexuses, mucosa and muscular layers	[14,29,30] [31]
Cat	nerve fibers in the myenteric ganglia and muscle layers	[19]
Sheep	nerve fibers in the myenteric ganglia and muscle layers	[19,33]
Ferret	nerve fibers in the myenteric ganglia and muscle layers	[19]
**Pancreas**		
Rat	endocrine parts of pancreas	[18]
Sheep	nerve fibers in the islet of Langerhans and small arteries neurons in the intrapancreatic ganglia	[32,33]

**Table 2:** The involvement of PACAP in the regulation of gastrointestinal disorders.

Organ	Species	Disease Model	References
Stomach	Rat	extrinsic denervation experimental ulcers	[7] [107]
	Pig	experimentally-induces hyperacidity diabetes	[24] [11]
Small intestine	Rat	intestinal autotranslantation ischemia-reperfusion	[101] [102]
	Pig	diabetes	[11]
	Mouse	cold and warm preservation in the course of intestinal transplantation	[103]
Intestinal INT407 cells	Human	lipopolysaccharide (LPS) exposure and bacterial adherence (*Escherichia coli, Salmonella Typhimurium, Klebsiella pneumoniae, Enterococcus faecalis*) oxidative stress	[94] [104]
Duodenum	Pig	naproxen and indomethacin administration	[25,108]
Ileum	Mouse	acute ileitis subacute ileitis	[92] [93]
	Rat	a dysfunctional (atrophic) intestine	[106]
	Pig	zearalenone intoxication	[12]
Large intestine	Human	inflammatory bowel disease carcinoma Hirschsprung’s disease	[100] [14] [29]
Colon	Mouse	dextran sulphate sodium (DSS)-induced colitis acute *Campylobacter jejuni*-induced enterocolitis	[96,97,98] [99]
	Human	symptomatic diverticular disease drug-resistant ulcerative colitis malignant tumor	[30] [31] [15]
	Pig	chemically-induced inflammation proliferative enteropathy axotomy of caudal colonic nerves diabetes	[10] [10] [10] [11]
Colonic Caco-2 cells	Human	lipopolysaccharide (LPS) exposure and bacterial adherence (*Escherichia coli, Salmonella Typhimurium, Klebsiella pneumoniae, Enterococcus faecalis*)	[94]
HCT8 human colonic tumor cell	Pig	tumor	[109]
Liver	Pig	bisphenol A dietary exposure	[13]

## Data Availability

Not applicable.
